# Dietary supplementation with Lactium and L-theanine alleviates sleep disturbance in adults: a double-blind, randomized, placebo-controlled clinical study

**DOI:** 10.3389/fnut.2024.1419978

**Published:** 2024-06-17

**Authors:** Su Eun Lim, Ho Seok Kim, Siwoo Lee, Eun Young Kang, Jong-Hyun Lim, Byung-Yong Kim, Seon-Mi Shin, Younghwa Baek

**Affiliations:** ^1^KM Data Division, Korea Institute of Oriental Medicine, Daejeon, Republic of Korea; ^2^R&D Center, Chong Kun Dang Healthcare, Seoul, Republic of Korea; ^3^Department of Internal Korean Medicine, College of Korean Medicine, Semyung University, Jecheon, Republic of Korea

**Keywords:** nutritional supplements, Lactium, sleep disturbance, sleep quality, clinical trial

## Abstract

**Introduction:**

The use of natural products for the treatment of sleep disturbances is increasing owing to the side effects and limitations of traditional sleep therapy. Moreover, recent studies have shown a significant correlation between sleep quality and gut microbiota composition. This study aimed to assess the impact of LTC-022, a commercially available dietary supplement containing Lactium and L-theanine, on enhancing sleep quality.

**Methods:**

Forty participants experiencing sleep discomfort were enrolled in a double-blind randomized controlled trial, wherein they received LTC-022 or a placebo orally for 8 weeks. The effects of treatment on sleep quality were assessed using the Pittsburgh Sleep Quality Index and Insomnia Severity Index. To comprehensively evaluate changes in sleep patterns, various parameters were evaluated, including the time in bed (TIB), total sleep time (TST), sleep onset latency (SOL), sleep efficiency (SE), wake after sleep onset (WASO) counts, and bedtime. These parameters were derived from daily sleep logs recorded over the 8-week study period, categorized into weekdays and weekends. Stool samples were analyzed for microbiome composition. The V4 region of bacterial 16S rRNA genes was amplified using specific primers (515F and 806R) and targeted for analysis. Microbial diversity, including operational taxonomic units, the Shannon and Chao indices, the Firmicutes/Bacteroidetes (F/B) ratio, and the variety of bacterial taxa, was assessed.

**Results:**

No significant differences were observed in sleep quality and insomnia scale characteristics between the two groups. In-depth analysis using sleep diaries showed that WASO counts after 8 weeks and bedtime after 4 weeks showed significant differences between the LTC-022 and control groups. In the LTC-022 group, significant differences were observed in the increase in TST, decrease in SOL, increase in SE, decrease in WASO counts, and earlier bedtime. Microbiome analysis revealed that the abundance of the genera *Blautia* and *Ruminococcus* increased in fecal samples from the LTC-022 group.

**Conclusion:**

These results suggest that continuous LTC-022 intake has a beneficial effect on maintaining sleep duration and an appropriate bedtime. Additionally, changes in the gut microbiota may be linked to changes in sleep patterns resulting from the consumption of Lactium and L-theanine.

**Clinical trial registration:**

https://cris.nih.go.kr/cris/search/detailSearch.do/22841, KCT0007750.

## 1 Introduction

Sleep disturbances are a common health problem in the modern 24-h society (1). During the recent coronavirus disease pandemic, the overall prevalence of sleep disorders was approximately 36%, and two people per week were reportedly experience low sleep quality (2, 3). Poor sleep due to short sleep time and low sleep quality can increase the risk of physical and mental disorders—including cardiovascular disease, cancer, and depression (4, 5)—and decrease the quality of life (6). A recent worldwide survey reported large variations in the prevalence of insomnia in the general population (2.3–25.5%), as well as a very close association between insomnia and mental health in several regions (7). However, half of those with sleep discomfort neglect or fail to recognize their issues (8). Additionally, only 20% of those with insomnia and 10% with excessive sleepiness are properly diagnosed and treated (9).

Interestingly, 41% of Koreans report poor sleep quality due to the influence of various social, environmental, and genetic factors (10). Meanwhile, the proportion of people visiting hospitals owing to sleep disturbances has been increasing by 8% per year (11). The self-management of sleep disturbances may promote interest in taking health supplements. One in five individuals in the general population reported taking natural product-based sleep aids (12). However, although some of these products have shown promising results, high-quality clinical trials to establish the safety and efficacy of these supplements are still lacking (13).

“Lactium” is a synthetic derivative of alpha-s1 casein hydrolysate containing the alpha-casozepine peptide, which is one of the main components of milk protein (14). Previous animal experiments have shown that Lactium improves stress-induced sleep problems by exerting calming and anxiolytic effects via the activation of gamma-aminobutyric acid (GABA) receptors in the central nervous system (15). GABA is well known for its sleep-promoting properties, and GABA-producing bacteria have the potential to improve sleep and treat sleep disorders (16). The rationale for consuming Lactium during sleep is rooted in the observation that milk has a calming effect on infants. Additionally, αS1-casein, as demonstrated in animal studies, can ameliorate sleep disorders induced by anxiety and stress, demonstrating its efficacy in alleviating stress-related symptoms (17). Objective assessments of actigraphy indices revealed more significant increases in sleep efficiency and overall sleep quality in the experimental group than that in the control group (17, 18). Moreover, a multinational study reported significant improvements in stress levels and sleep quality within 1 month after supplementation with Lactium^®^, with 80% of the participants reporting satisfaction with its effects (19). L-theanine, chemically known as gamma-glutamyl ethyl amide, is a unique non-protein amino acid that was first discovered in green tea in the mid-20th century. This supplement has proven antioxidant and anxiolytic effects (20). Previous research has shown that L-theanine positively improves the quality of sleep in adults, and children by regulating neurotransmitters and inhibiting excitatory neurons (21). This suggests that it can be consumed as a natural supplement to enhance sleep quality across all age groups.

Research focusing on the correlation between sleep and the gut microbiota is increasing. Recent studies have revealed that the gut microbiota not only influences normal sleep but also impacts abnormal sleep patterns and durations, as evidenced by the increasing focus on the gut–brain axis (22). The bidirectional relationship between sleep and the gut microbiota has been elucidated in various studies, highlighting the finding that improving sleep can positively affect the composition of the gut microbiome. In a previous study, the intake of L-theanine was associated with an increase in beneficial bacteria, such as *Lactobacillus*, and a decrease in harmful bacteria, such as *Clostridium*, in broiler chickens (23). Currently, no clinical trials have directly investigated the effects of L-theanine intake on gut health in participant. Our study represents one of the first attempts to explore this potential health benefit in participant, building on preliminary findings from animal models. Recognizing this gap in the existing literature, our aim was to provide a foundational study that could encourage further clinical research in this domain. This suggests that the consumption of sleep-promoting agents may modulate the gut microbiome, indicating a potentially beneficial role of Lactium and L-theanine in promoting better sleep.

The present study sought to evaluate the effect of a commercially available dietary supplement (tentatively named LTC-022), containing Lactium and L-theanine as its main ingredients, on improving sleep quality. Furthermore, this study aimed to explore changes in the gut microbiome resulting from the consumption of LTC-022.

## 2 Materials and methods

### 2.1 Study design and procedure

This was a single-center, double-blind, randomized, placebo-controlled clinical trial. Eligible participants were administered LTC-022 or a placebo daily for 8 weeks and visited the hospital four times (screening, allocation, baseline measurement, an interim visit at 4 weeks from the start of the intervention, and a close-out visit at 8 weeks after the intervention) for efficacy and safety assessments. The participants were instructed to orally ingest one packet (one tablet of Lactium and one tablet of L-theanine) per day with water 1 h before bedtime for 8 weeks. The placebo, having the same color and size as LTC-022, contained lactose and dextrin as the main ingredients, and it was ingested in the same manner. The trial was approved by the Institutional Review Board of Semyung University Oriental Medicine Hospital (number: SMJOH-2022-07) and retroactively registered in the Clinical Research Information Service (number: KCT0007750^1^).

### 2.2 Participants

**Figure 1:**
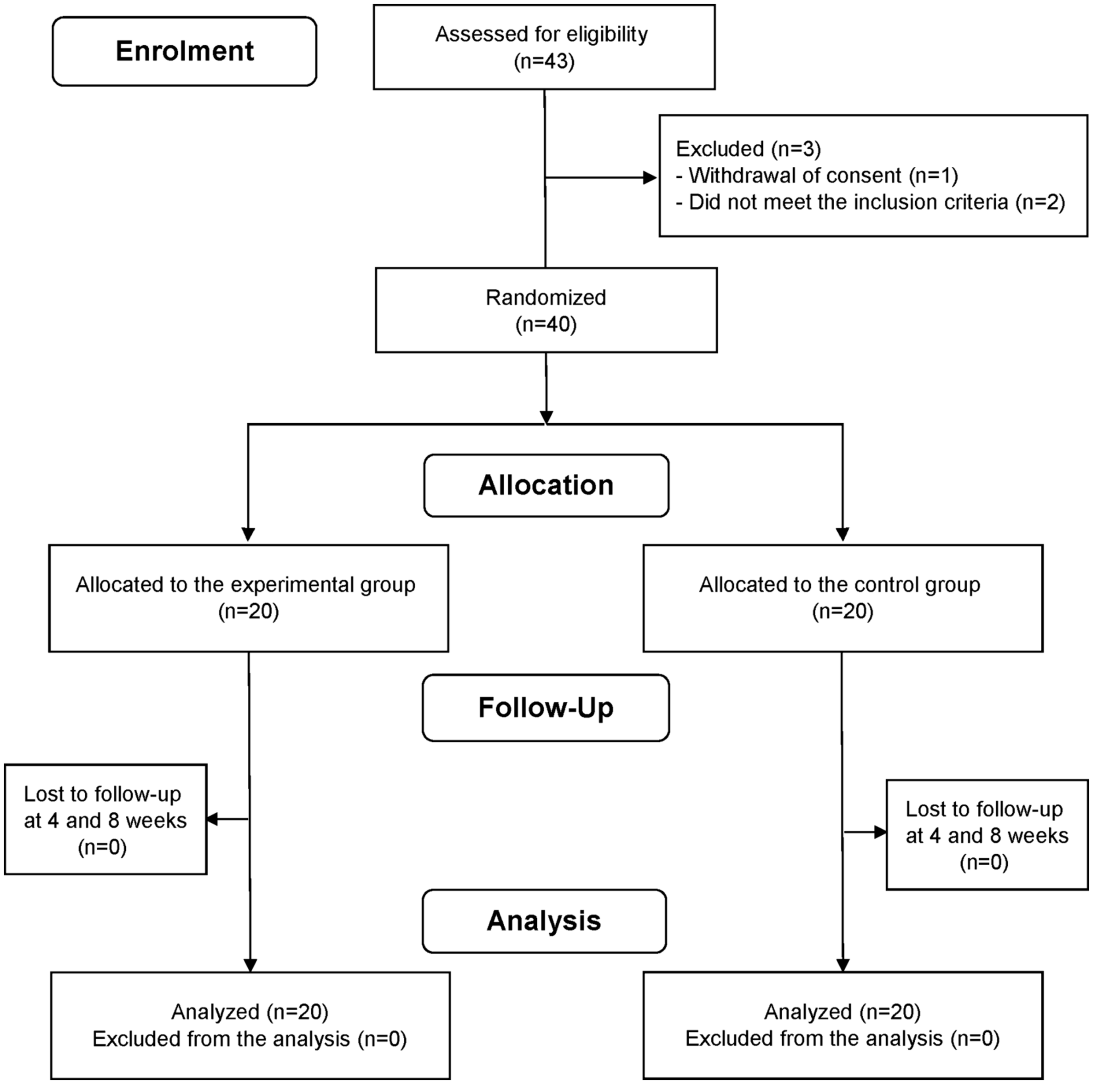
Study protocol.

This study, conducted between November 2022 and May 2023, involved adults aged 30–59 years who reported that they experienced sleep disturbances. The choice of the age group in our study was based on several considerations. While it is true that life events such as having young children or experiencing menopause can significantly affect sleep patterns, we aimed to focus on a broad adult population. In the case of the elderly, sleep problems may manifest differently due to aging. By selecting the age range of 30 to 59 years, we aimed to capture a diverse group of individuals who are likely to encounter various life events and stressors. To exclude individuals with severe sleep disturbances, the study only included individuals with a Pittsburgh Sleep Quality Index (PSQI) score of >5 points and an Insomnia Severity Index (ISI) score of ≥8 but ≤21 points. The eligibility criteria for the participants are presented in Supplementary Table S1. The sample size was calculated as 40 (20 each in the experimental and control groups), considering a 10% dropout rate. In accordance with a previous study, the sample size was estimated based on the following statistical parameters: an assumed statistical power of 80%; a significance level (α) of 0.05 for two-sided, two-sample, equal variance *t*-tests; a difference of 2.4 in the amount of change in PSQI scores between the LTC-022 and placebo groups; and a standard deviation of 2.5 (24). A total of 43 participants were registered for screening. Two participants were excluded owing to a PSQI score < 5 and an ISI score > 21, and one participant withdrew their consent before randomization. Therefore, 40 participants were included in the randomization step (Figure 1). Data on experimental adjuvants administered to participants were collected at baseline and at weeks 4 and 8.

### 2.3 Interventions

Participants received either LTC-022 or placebo supplementation for 8 weeks. The main ingredients of LTC-022 are Lactium and L-theanine; these two functional ingredients have been individually approved for use in maintaining sleep health and recognized by the Ministry of Food and Drug Safety in Korea (Lactium: No. 2020-2, L-theanine: No. 2011-11) (25). The form of LTC-022 used in the present study is commercially available in Korea and was supplied by the Chronic Kidney Disease Healthcare Group (Korea). LTC-022 consists of individual tablets containing Lactium and L-theanine. The Lactium tablet was a 500 mg white tablet containing 60% Lactium, 15% crystalline cellulose, 12.5% dextrin, and other ingredients. The L-theanine tablet was a 700 mg gray tablet containing 29.16% L-theanine, 30% maltodextrin, 19.789% crystalline cellulose, and other ingredients. The participants were instructed to orally ingest one packet (one Lactium tablet and one L-theanine tablet) per day with water 1 h before bedtime for 8 weeks. The dose used in this trial was the recommended dose for these commercially available products.

### 2.4 Outcomes

The primary endpoint was sleep quality, as measured using the PSQI (26). The PSQI is used to assess sleep quality and disturbances based on sleep habits in the previous month. It is widely applied in clinical and non-clinical practice with adequate validity. The PSQI consists of 19 items categorized into 7 domains, with total scores ranging from 0 to 21 points. A score ≥ 5 points indicates poor sleep quality.

The secondary endpoint was insomnia symptoms, which were measured using the ISI (27). The ISI consists of 7 items, with total scores ranging from 0 to 28 points. Higher scores indicate more severe insomnia symptoms (27). In addition, an in-depth analysis of sleep profiles was performed based on data obtained using continuous daily sleep diary entries. The template of the sleep diary was designed in accordance to a previous study (28), after modifying some of the contents. The sleep diary variables were time in bed (TIB), total sleep time (TST), sleep on latency (SOL), sleep efficiency (SE), wake after sleep onset (WASO) counts, and bedtime. In an in-depth analysis of sleep patterns, 5 days of the week and 2 days of the weekend were examined to investigate the differences in sleep time patterns between sleep during the week and sleep on weekends.

### 2.5 DNA extraction and microbiome analysis

Stool samples were collected twice at baseline, prior to LTC-022 consumption and at 8 weeks following the completion of the intake period. Samples for microbiome analysis were collected using a specialized stool kit (NBG-1C; NobleBio Inc., Seoul, Korea). Genomic DNA was extracted using the Omega Mag-Bind DNA Prep Kit (Omega Bio-tek Inc., Norcross, GA, United States) according to the manufacturer’s instructions. Amplification of the V4 region of the bacterial 16S rRNA genes was performed using the primers 515F (TCGTCGGCAGCGTCAGATGTGTATAAGAGACAGGTGCCAGCMGCCGCGGTAA) and 806R (GTCTCGTGGGCTCGGAGATGTGTATAAGAGACAGGGACTACHVGGGTWTCTAAT). These primers were selected to specifically target the V4 region of bacterial 16S rRNA genes. Subsequently, the amplified DNA fragments were used for downstream microbiome analyses. The polymerase chain reaction (PCR) conditions were as follows: initial denaturation at 95°C for 3 min followed by 25 cycles of denaturation at 95°C for 30 s, annealing at 55°C for 30 s, and extension at 72°C for 30 s, with a final extension phase at 72°C for 5 min. The PCR products were sequenced using an Illumina i-Seq 100 system (Illumina, Inc., San Diego, CA, United States) at the R&D Center of Chong Kun Dang Healthcare. The sequence data were subjected to clustering of operational taxonomic unit (OTU) representative sequences at 98% similarity using a pipeline developed in the CLC Genomics Workbench 22.0 (QIAGEN, Aarhus, Denmark) at the R&D Center of Chong Kun Dang Healthcare. Taxonomic richness was determined using the SILVA 115 database as reference (29).

### 2.6 Demographic data

Demographic information (sex and age), anthropometric measurements (body mass index), lifestyle factors (smoking and alcohol consumption status), and vital signs were included as covariates. Anthropometric variables were measured using digital scales, while all other data were collected using structured questionnaires.

### 2.7 Safety indices and compliance

The safety of the treatment was assessed based on alanine aminotransferase (ALT) and aspartate aminotransferase (AST) levels (hepatic function). Both safety indices were examined during the screening visit and at 8 weeks, after completion of the intervention. Compliance was calculated using the following formula: the number of experimental adjuvants actually used divided by the number of experimental adjuvants that should have been used during the study period. The use of experimental adjuvants was further assessed based on daily sleep diary entries.

### 2.8 Randomization

The randomization code was generated as four block sizes in a 1:1 ratio for the experimental and control groups by the sponsor or designated manager before the start of the study. Randomization was conducted by assigning sequential numbers to eligible participants based on the results of the screening test. The investigators and participants were blinded to the group allocation and the items consumed by the participants. During the study period, double-blinding of the investigators and participants was maintained. The paper containing information on group allocation was kept in a sealed envelope until unblinding. In the event of emergency unblinding, the unblinding envelope was to be provided to the investigator.

### 2.9 Statistical analysis

Two-sided statistical analyses were conducted, with the significance level set at 5%. In the full analysis set, the intent-to-treat approach was utilized to analyze the endpoints. In the event of discontinuation of observation and treatment, the cause was identified and analyzed using the last-observation-carried-forward method. The baseline demographic and clinical characteristics were summarized using descriptive statistics. Categorical variables are presented as numbers and percentages and were analyzed using the chi-square test. Continuous variables are expressed as mean ± standard deviation (SD) and were analyzed using two-sample *t*-tests. The primary and secondary endpoints were analyzed using paired *t*-tests of variance, with values at 4 and 8 weeks after initiation of the intervention relative to the baseline values as the dependent variables. Secondary endpoints were analyzed using the same approach. A two-sample *t*-test was performed to examine the difference between groups at each visit. A paired *t*-test was conducted to examine the difference according to the visit in each group. Additionally, an analysis of covariance was performed, setting the baseline value as a covariate to more accurately estimate the difference in treatment effects between the two groups. A two-way repeated-measures analysis of variance was used to explore the interaction effect between time (baseline, Week 4, and Week 8) and group (LTC-022 and control). The results of the gut microbiome analysis were presented using GraphPad Prism (version 6.0, GraphPad Software Inc., United States) and are displayed as mean ± SD. Statistical significance between different groups was analyzed using one-way analysis of variance. The correlation between the gut microbiota and obesity-related indicators was analyzed using Pearson’s correlation. Significance was validated at *p* < 0.05.

## 3 Results

The demographic characteristics of the 40 participants enrolled at the time of randomization are presented in Table 1. The average ages were 48.2 and 47.4 years in the LTC-022 and control groups, respectively. In the LTC-022 and control groups, 1 man and 19 women participated in each group, and no differences were observed between the two groups (Table 1).

**Table 1:** Participant characteristics.

Characteristic	Total	LTC-022 (*n* = 20)	Control (*n* = 20)	*p*-value
**Sex, *n* (%)**				
Male	2 (5.0)	1 (5.0)	1 (5.0)	1.000
Female	38 (95.0)	19 (95.0)	19 (95.0)	
Age, yr. (SD)	47.8 (6.8)	48.2 (6.7)	47.4 (7.1)	0.176
BMI, kg/m^2^ (SD)	23.3 (2.5)	23.3 (2.6)	23.3 (2.4)	0.954
**Drinking, *n* (%)**				
Yes	18 (45.0)	10 (50.0)	8 (40.0)	0.751
No	22 (55.0)	10 (50.0)	12 (60.0)	
**Smoking, *n* (%)**				
Yes	3 (7.5)	2 (10.0)	1 (5.0)	1.000
No	37 (92.5)	18 (90.0)	19 (95.0)	
**Safety indices**				
AST, U/L (SD)	20.60 (6.69)	22.20 (7.56)	19.00 (5.42)	0.132
ALT, U/L (SD)	16.78 (8.14)	18.45 (8.91)	15.10 (7.12)	0.197

SD, standard deviation; BMI, body mass index; AST, aspartate aminotransferase; ALT, alanine aminotransferase.

*p*-values were obtained using chi-square tests for categorical variables and two-sample *t*-tests for continuous variables.

Differences in sleep quality and insomnia scale characteristics are shown in Table 2. In the LTC-022 group, the PSQI scores (SD) were 6.75 (2.34) after 4 weeks and 6.55 (2.48) after 8 weeks, which were significantly lower than the baseline score of 9.70 (2.43) (all *p*-value < 0.05 by paired *t*-test). However, no significant interaction effect between time and group was observed after the intervention (p-interaction = 0.621). In the LTC-022 group, the ISI score decreased significantly from a baseline score of 13.00 (4.3) to 8.60 (3.78) and 7.25 (3.68) after 4 and 8 weeks, respectively (all *p*-value < 0.001 by paired *t*-test). The control group showed similar results, with scores decreasing from a baseline of 14.2 (3.16) to 10.75 (4.61) and 9.4 (5.71) after 4 and 8 weeks, respectively. However, no significant interaction effect between time and group was observed after the intervention (p-interaction = 0.676) (Table 2).

**Table 2:** Differences in sleep status between LTC-022 and control groups Sleep quality was measure by Pittsburgh Sleep Quality Index, and Insomnia severity was by Insomnia Severity Index.

Variables	LTC-022	Control	*p*-value	p-interaction^†^
**Sleep quality**				
Baseline	9.7 (2.43)	10.8 (2.86)	0.198^a^	0.621
Week 4	6.75 (2.34)^*^	7.9 (2.83)^*^	0.340^b^	
Week 8	6.55 (2.48)^*^	6.95 (2.96)^*^	0.847^b^	
**Insomnia severity**				
Baseline	13.0 (4.30)	14.2 (3.16)	0.322^a^	0.676
Week 4	8.60 (3.78)^*^	10.75 (4.61)^*^	0.211^b^	
Week 8	7.25 (3.68)^*^	9.4 (5.71)^*^	0.282^b^	

^†^ Two-way repeated-measures ANOVA analysis (Time [Baseline, Week 4 and Week 8] × Group [LTC-022 and Control]).

^*^ *p*-value < 0.05 are based on paired *t*-tests between the parameters “Week” and “baseline” in each group.

a *p*-values are based on two-sample *t*-tests between the LTC-022 and Control groups in the baseline.

b *p*-values are based on ANCOVA analysis adjusted for baseline values of each outcome variable.

The sleep monitoring results from the subjective sleep diary are presented in Table 3. On weekdays, significant differences between the LTC-022 and control groups were found in WASO counts (*p*-value = 0.01) after 8 weeks and in bedtime (*p*-value = 0.021) after 4 weeks. A significant interaction effect between time (baseline, Week 4, and Week 8) and group (LTC-022 and Control group) was also observed for WASO counts (p-interaction = 0.007) and was marginally observed for bedtime (p-interaction = 0.083) across the study duration. Additionally, significant results were found in the TST, SE, WASO counts, and bedtime after 4 and 8 weeks in the LTC-022 group. The *p*-values were < 0.05 for both 4 weeks and 8 weeks based on paired t-tests (TST, 7.3-min increase; SE, 0.36% increase; WASO counts, 0.31 decrease; and bedtime, 5-min decrease). The weekend results showed significant increases in the TST at 8 weeks in the LTC-022 group, with a difference of 14.31 min compared to the TST at 4 weeks. Bedtime was also significantly reduced by 19 min at 8 weeks compared with that at 4 weeks in the LTC-022 group (Table 3). No adverse events were reported. In the LTC-022 group, no significant changes were observed in the AST and ALT levels after the intervention compared to baseline, and no participant experienced an elevation of values to more than 1.5 times the upper limit of the reference range.

**Table 3:** Comparison of sleep diary data between the LTC-022 and control groups.

Variables	Weekday				Weekend			
	LTC-022	Control	*p*-value	p-interaction^†^	LTC-022	Control	*p*-value	p-interaction^†^
**TIB (min)**								
Baseline	456.70 (57.70)	455.35 (72.05)	0.949^a^	0.864	453.88 (57.50)	453.33 (73.77)	0.980^a^	0.656
Week 4	458.28 (47.64)	450.09 (76.19)	0.744^b^		476.27 (74.51)	474.72 (116.3)	0.839^b^	
Week 8	465.09 (55.82)	457.17 (112.63)	0.767^b^		482.88 (65.18)	462.29 (81.43)	0.345^b^	
**TST (min)**								
Baseline	392.40 (58.04)	400.39 (49.73)	0.648^a^	0.367	401.13 (52.49)	412.38 (56.38)	0.523^a^	0.300
Week 4	412.45 (45.41)^*^	397.70 (53.69)	0.143^b^		428.94 (82.58)	421.71 (86.08)	0.444^b^	
Week 8	419.75 (53.98)^*^	407.86 (80.42)	0.404^b^		443.25 (65.26)^*^	414.50 (59.31)	0.123^b^	
**SOL (min)**								
Baseline	64.30 (40.53)	54.96 (59.80)	0.570^a^	0.355	52.75 (41.97)	40.96 (52.29)	0.441^a^	0.359
Week 4	45.83 (31.84)^*^	52.39 (66.08)	0.135^b^		47.33 (41.30)	53.01 (69.36)	0.280^b^	
Week 8	45.34 (41.67)	49.30 (58.81)	0.499^b^		39.63 (34.78)	47.79 (58.65)	0.273^b^	
**SE (%)**								
Baseline	86.04 (8.58)	88.93 (10.27)	0.346^a^	0.222	88.75 (8.46)	91.72 (8.49)	0.281^a^	0.349
Week 4	90.14 (6.17)^*^	89.34 (10.32)	0.124^b^		89.91 (8.23)	90.21 (10.17)	0.528^b^	
Week 8	90.50 (8.06)^*^	90.56 (8.97)	0.527^b^		91.81 (6.51)	90.65 (10.09)	0.253^b^	
**WASO (counts)**								
Baseline	2.42 (1.49)	1.66 (0.74)	0.053^a^	0.007	1.95 (0.97)	1.89 (0.80)	0.824^a^	0.601
Week 4	1.62 (1.68)^*^	1.55 (0.71)	0.119^b^		1.50 (1.67)	1.79 (0.94)	0.499^b^	
Week 8	1.31 (1.83)^*^	1.63 (0.96)	**0.010**^b^		1.60 (2.55)	1.53 (0.99)	0.926^b^	
**Bedtime (hh:mm)**								
Baseline	24:33 (59.66)	24:22 (67.06)	0.605^a^	0.083	24:43 (53.01)	24:28 (85.43)	0.524^a^	0.193
Week 4	24:06 (48.32)^*^	24:25 (66.72)	**0.021**^b^		24:20 (66.33)	24:25 (84.43)	0.503^b^	
Week 8	24:01 (56.44)^*^	24:25 (68.58)	0.090^b^		24:01 (62.02)^*^	24:29 (66.34)	0.055^b^	

^†^ Two-way repeated-measures ANOVA analysis (Time [Baseline, Week 4 and Week 8] × Group [LTC-022 and Control]).

^*^ *p*-value < 0.05 are based on paired *t*-tests between the parameters “Week” and “baseline” in each group.

a *p*-values are based on two-sample t-tests between the LTC-022 and Control groups in the baseline.

b *p*-values are based on ANCOVA analysis adjusted for baseline values of each outcome variable.

To assess the improvement in gut microbiota clustering associated with the efficacy of sleep enhancement, we examined the changes in the microbiota before and after the intake of Lactium and L-theanine, which are known for their sleep-improving effects.

**Figure 2:**
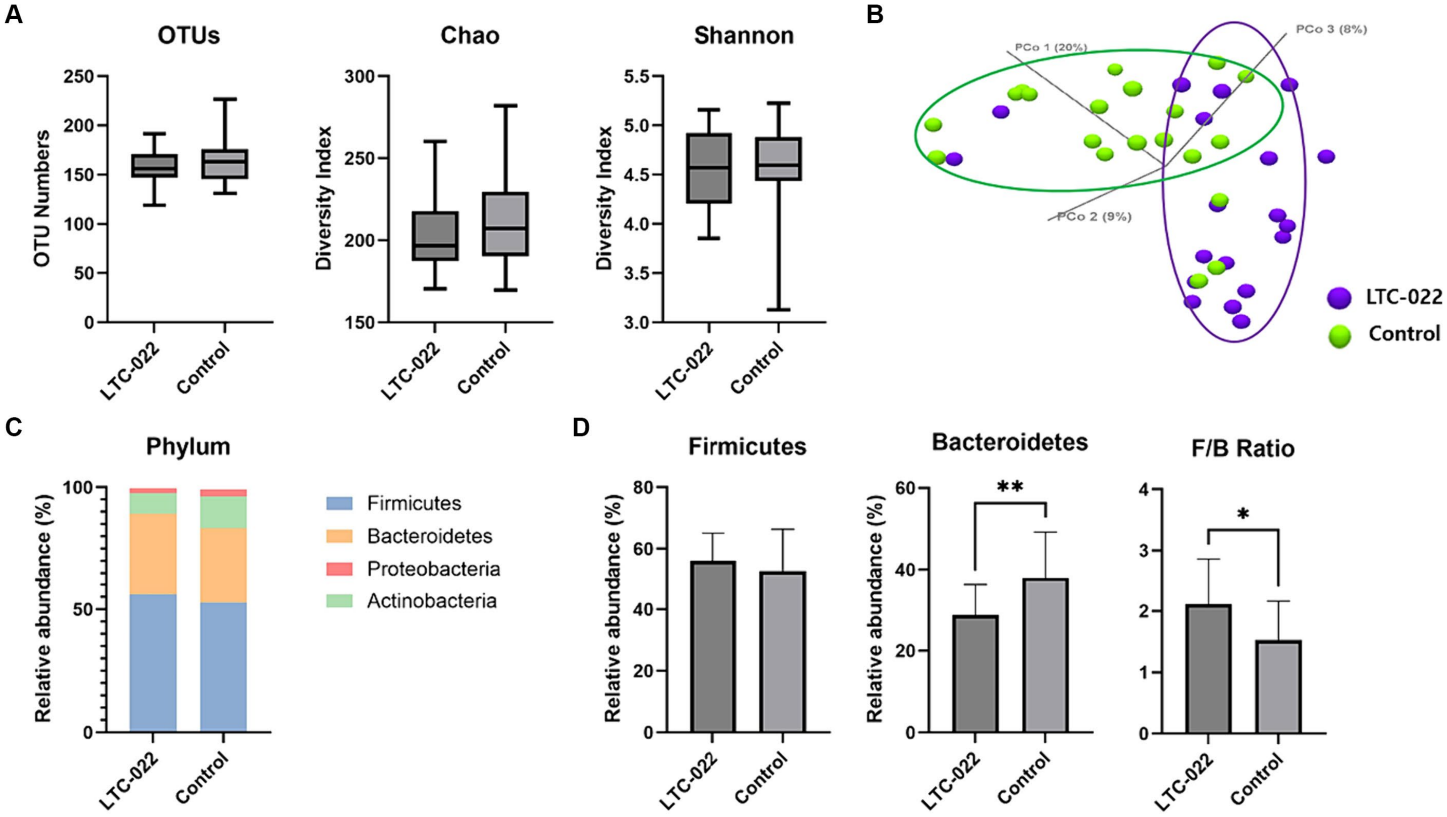
Effects of Lactium and L-theanine intake on microbiome composition. Presenting the numerical values of Operational Taxonomic Units (OTUs), Chao, and Shannon indices **(A)** between the LTC-022 and the control groups, while explaining alpha-diversity; demonstrating beta-diversity **(B)**, where purple represents the LTC-022 group and green represents the control group; intestinal bacteria taxonomic profiling at the phylum **(C)** level for the LTC-022 and control groups; and the relative abundance of the bacterial phylum and Firmicutes/Bacteroidetes ratio in the fecal samples of the LTC-022 and control groups **(D)**.

The alpha diversity of each group was evaluated using OTUs and the Shannon and Chao indices. The results showed no significant difference in the changes in richness represented by OTUs and the Shannon and Chao indices between the LTC-022 and control groups after the intervention (Figure 2A). Based on the Bray–Curtis dissimilarity using principal coordinates analysis, there were no significant differences in beta diversity between the LTC-022 and control groups (Figure 2B).

All stool samples contained five phyla: Firmicutes, Bacteroidetes, Actinobacteria, and Proteobacteria (each had a relative abundance <1%) (Figure 2C). Among them, Firmicutes and Bacteroidetes constituted the majority (Figure 2D). Significant differences were observed in the Firmicutes/Bacteroidetes (F/B) ratio and abundance of Bacteroidetes between the LTC-022 and control groups.

After the intervention, significant differences were observed in the abundance of specific microbes between the LTC-022 and control groups, namely, *Bifidobacterium*, *Prevotella*, *Erysipelatoclostridium*, and *Megamonas* (Table 4).

**Table 4:** Microbial changes after the intervention in the LTC-022 and control groups.

Genus	LTC-022	Control	*p*-value
Bifidobacterium	5.13 (4.79)	9.74 (6.62)	0.041
Prevotella	13.23 (12.45)	3.09 (6.53)	0.027
Erysipelatoclostridium	0.18 (0.62)	0.29 (0.41)	0.024
Megamonas	1.96 (4.08)	0.62 (2.50)	0.018

*p*-values are based on two-sample *t*-tests between the LTC-022 and control groups.

Data are expressed as mean (±standard deviation).

Furthermore, the LTC-022 group showed an increase in the abundance of *Blautia*, *Hungatella*, and *Ruminococcoides*, and a decrease in the abundance of *Bilophila*, *Clostridium*, and *Holdemania*, whereas these changes were not observed in the control group (*p* < 0.05) (Table 5).

**Table 5:** Microbial changes in the LTC-022 group before and after the intervention.

LTC-022			
Genus	Baseline	Week 8	*p*-value
*Bilophila*	0.17 (0.18)	0.10 (0.06)	0.046
*Blautia*	6.59 (3.40)	7.43 (2.94)	0.031
*Clostridium*	0.47 (0.86)	0.22 (0.55)	0.027
*Holdemania*	0.02 (0.02)	0.01 (0.01)	0.045
*Hungatella*	0.27 (0.24)	0.60 (0.79)	0.027
*Ruminococcoides*	0.02 (0.09)	1.08 (2.44)	0.037
*Ruminococcus*	2.01 (2.12)	3.54 (3.37)	0.003
*Sutterella*	1.73 (1.74)	0.99 (0.77)	0.017

*p*-values are based on two-sample *t*-tests.

Data are expressed as mean (±standard deviation).

In contrast, the control group showed an increase in the abundance of *Anaerobutyricum*, *Bifidobacterium*, *Collinsella*, *Eubacterium*, and *Odoribacter*, and a decrease in the abundance of *Faecalibacterium*, *Parasutterella*, and *Prevotella* (*p* < 0.05) (Table 6). Additionally, after the intervention, both the control and LTC-022 groups exhibited similar trends, with an increase in the abundance of *Ruminococcus* and a decrease in that of *Sutterella* (*p* < 0.05).

**Table 6:** Microbial changes in the control group before and after the intervention.

Control			
Genus	Baseline	Week 8	*p*-value
*Anaerobutyricum*	1.07 (0.81)	1.50 (2.05)	0.039
*Bifidobacterium*	5.34 (3.39)	9.71 (6.59)	0.003
*Collinsella*	1.96 (1.88)	2.93 (2.71)	0.045
*Eubacterium*	0.13 (0.17)	0.29 (0.45)	0.049
*Faecalibacterium*	15.91 (8.56)	11.95 (6.88)	0.008
*Odoribacter*	0.15 (0.13)	0.26 (0.28)	0.044
*Parasutterella*	0.65 (1.06)	0.31 (0.49)	0.026
*Prevotella*	6.52 (9.91)	3.09 (6.53)	0.049
*Ruminococcus*	2.13 (2.36)	2.97 (3.33)	0.047
*Sutterella*	1.01 (1.13)	0.70 (0.84)	0.017

*p*-values are based on two-sample *t*-tests.

Data are expressed as mean (±standard deviation).

## 4 Discussion

This study investigated the safety and efficacy of LTC-022 intake in 40 adults experiencing sleep discomfort. Participants were randomly assigned to double-blind and placebo groups for an 8-week intervention period. Questionnaire-based sleep indicators and intestinal microbes were analyzed. In this study, Lactium and L-theanine, the main components of LTC-022, improved sleep quality and insomnia symptoms. However, similar trends were observed in both groups, showing no difference between them. However, in the sleep analysis reflecting real-life sleep conditions, WASO counts decreased and bedtimes were earlier in the LTC-022 group than that in the control group. The variables that showed differences only in the LTC-022 group were the TST, SE, and SOL.

This randomized controlled trial was designed to assess the clinical efficacy of LTC-022, which comprises Lactium and L-theanine as the main ingredients, on sleep quality. PSQI and ISI scores were measured on a subjective symptom scale, showing progressive improvement with LTC-022 consumption. However, no significant differences in PSQI and ISI scores were observed between the LTC-022 and control groups. The lack of an apparent effect of Lactium may be attributed to the small sample size (18). However, despite its small sample size, this study remains valuable due to its high compliance, averaging ≥95%. Nevertheless, further research is warranted. This study is significant in evaluating improvements in sleep quality using self-report questionnaires and sleep diaries, which are subjective methods. Previous studies have shown that sleep diaries have a high degree of agreement with activation measurements and sleep evaluation (30). Furthermore, the study confirmed that sleep efficiency—as measured using a sleep diary—and activity significantly improved in the intervention group compared to the control group (17). Therefore, this study prepared a sleep journal for healthy adults from the general population who experience sleep discomfort.

WASO counts decreased and bedtime shifted to an earlier time in the LTC-022 group after 4 and 8 weeks of intervention, respectively, compared to the control group. It was observed that these variables showed different changes between groups over time. In addition, a significant difference was observed in the LTC-022 group during weekdays, where TST increased, SE increased, WASO counts decreased, and bedtime was earlier at 8 weeks compared to that at 4 weeks. Sleep-related variables during weekdays were found to have more significant differences than those during weekend days, which typically mirrors weekday sleep patterns well (31). The component characteristics of LTC-022 may contribute to nerve stability before bedtime, resulting in an increase in TST, SE, and WASO counts. Furthermore, over the weekend, the LTC-022 group showed increased TST and an earlier bedtime after 8 weeks. Consistent with our findings, previous surveys using sleep journals showed that SE increased by approximately 10% after 4 weeks, compared to 2 weeks, improving sleep quality when Lactium-based health supplements were administered for a long time (17). In the Lactium study, there was a tendency for WASO to decrease, but no significant increase in sleep time was observed (17). In the L-theanine study, sleep time was increased by reducing sleep latency (32). Based on these two studies, we concluded that the combination of Lactium and L-theanine components resulted in a decrease in the number of WASO counts and an increase in bedtime.

The association between the microbiome and sleep has been established in numerous studies (33). In this study, we investigated the changes in the gut microbiome, focusing on microbial balance, when consuming Lactium and L-theanine. Significant trends were observed in the LTC-022 group, indicating an increase or decrease in certain microbes during gut microbiome analysis. The gut microbe *Blautia*, which is reported to increase with better sleep quality, increased in abundance in the LTC-022 group (34). Additionally, *Holdemania*, another microbe negatively correlated with sleep, showed a similar trend of significant reduction in post-intake levels, consistent with previous research findings (22). We also observed a tendency for a higher F/B ratio in the LTC-022 group, which is known to be positively correlated with sleep quality, seemingly because of a greater relative abundance of *Blautia* and *Ruminococcus* (Firmicutes) and lower proportions of *Prevotell*a (Bacteroidetes) in individuals with superior sleep quality (34).

*Ruminococcus* and *Sutterella*, which showed significant changes in both the LTC-022 and control groups, exhibited a consistent pattern. In both groups, the abundance of *Ruminococcus* increased significantly, whereas that of *Sutterella* decreased. In particular, *Ruminococcus* exhibited significant changes as a producer of short-chain fatty acids, indicating notable alterations in beneficial gut microbes (35). Thus, the abundance of beneficial bacteria, such as *Ruminococcus,* increased in both groups.

This study examined the correlation between sleep discomfort and the gut microbiome after the intake of Lactium and L-theanine, which are known to aid sleep. Variations were observed in the microbial changes, some aligning with previous research and others showing opposite trends. These findings suggest that the intake of Lactium and L-theanine may influence changes in microbial balance. Although further research and confirmation are necessary, these results support the correlation between sleep and the gut microbiome. However, the absence of significant changes in microbial alpha and beta diversity may be attributed to the non-implementation of certain dietary restrictions. Future studies may benefit from restricting the intake of dietary components that could influence the gut microbiome, such as probiotics, to assess changes in the gut microbiome more accurately.

A strength of this randomized, double-blind, placebo-controlled trial is the thorough investigation using a sleep diary, and the fact that it was conducted without any dropouts. Additionally, the study duration of 8 weeks was long enough to observe the improvement effect. Furthermore, measures were in place to ensure the safety of the participants and manage compliance, including a 5-day absence period after screening. This study evaluated the safety of the treatment by monitoring the levels of ALT and AST, which indicate liver function. Two safety indices were assessed during the examination visit and again 8 weeks after the intervention. Moreover, to overcome the uncertainty of treatment efficacy, the intervention period of this study was set to 8 weeks, at least twice as long as that used in previous studies (17, 19, 24) that investigated the short-term efficacy of the product. In addition, a study period of more than 8 weeks was established according to a previously-completed comparative study on Lactium and L-theanin (24). Sleep time was divided into weekdays and weekends, with bedtime and wake-up time being more flexible on weekends than on weekdays. This allowed for the analysis of sleeping habit patterns over 7 days (36).

One limitation of this study is the potential for recall bias associated with participants self-reporting their sleep time. Future studies should use objective measurements, such as a wearable activity tracker, to analyze sleep-related variables more accurately. Additionally, defining insomnia solely based on a PSQI score > 5 may have limited the accurate distinction between acute and chronic insomnia. Secondly, this study did not control for dietary intake and lifestyle factors. However, participants with milk allergies were excluded. Thirdly, the number of male participants were small (*n* = 1 in each group), leading to a sex bias; consequently, the effects of LTC-022 on male sex remains to be addressed. Further studies for male participants are needed.

## 5 Conclusion

This double-blind, randomized, placebo-controlled trial aimed to improve sleep quality in adults experiencing sleep discomfort using LTC-022 dietary supplements. Over an 8-week period, daily consumption of a dietary supplement (LTC-022) containing Lactium and L-theanine resulted in significant improvements in subjective sleep quantity and quality, including increased TST and SE, as well as reduced WASO counts and an earlier bedtime. Moreover, our results indicate that LTC-022 use for 8 weeks, compared to 4 weeks, is safe for individuals experiencing sleep disturbances and mild-to-moderate insomnia symptoms. Furthermore, we observed significant changes in the abundance of specific microbes, such as *Blautia* and *Holdemania*, in the LTC-022 group, suggesting that LTC-022 intake may influence the microbial balance related to sleep quality. Our findings contribute to the understanding of the relationship between sleep and the gut microbiome. Nonetheless, further exploration in this field is warranted.

## Data availability statement

The original contributions presented in the study are included in the article/Supplementary material, further inquiries can be directed to the corresponding authors.

## Ethics statement

The studies involving humans were approved by Semyung University Oriental Medicine Hospital. The studies were conducted in accordance with the local legislation and institutional requirements. The participants provided their written informed consent to participate in this study. Written informed consent was obtained from the individual(s) for the publication of any potentially identifiable images or data included in this article.

## Author contributions

SLi: Writing – original draft, Writing – review & editing, Data curation. HK: Formal analysis, Data curation, Writing – review & editing. SLe: Conceptualization, Project administration, Supervision, Writing – review & editing. EK: Writing – original draft. J-HL: Formal analysis, Writing – review & editing. B-YK: Project administration, Writing – review & editing. S-MS: Conceptualization, Investigation, Writing – review & editing. YB: Conceptualization, Project administration, Writing – review & editing.

## Footnotes

^1^ https://cris.nih.go.kr/cris/search/detailSearch.do/22841
