# Supplementary material for: Dietary supplementation with Lactium and L-theanine alleviates sleep disturbance in adults: a double-blind, randomized, placebo-controlled clinical study
Source: Front Nutr. 2024 Jun 17;11:1419978. doi: 10.3389/fnut.2024.1419978 (PMC11215043; doi:10.3389/fnut.2024.1419978)
Supplement: Supplementary file 1 [file Table_1.DOCX]

Supplement

Table 1. Eligibility criteria

| **Inclusion criteria** |
| --- |
| 1) Adults aged 30–59 years  2) Individuals with a Pittsburg Sleep Quality Index score of >5 points  3) Individuals with Insomnia Severity Index scores of ≥8 but ≤21 points  4) Individuals who will consent to participate in the study and submit an informed consent form prior to the start of the study |
| **Exclusion criteria** |
| 1) Individuals currently receiving treatment for severe cardiovascular, immune, respiratory, gastrointestinal/liver, biliary tract, renal, urinary, nervous, musculoskeletal, and infectious diseases and those with malignant tumors  2) Patients receiving medical or traditional Korean medicine treatment or care for sleep disturbance within 4 weeks from Visit 1 (screening)  3) Patients receiving treatment for generalized anxiety disorder, post-traumatic stress disorder, obsessive-compulsive disorder, or major depressive disorder based on the Diagnostic and Statistics Manual of Mental Disorders, 5^th^ Edition criteria  ⓐ Individuals with a Patient Health Questionnaire-9 score of ≥10 points  ⓑ Individuals with a Generalized Anxiety Disorder-7 score of ≥5 points  4) Patients with current or previous history of mental illness (e.g., schizophrenia and bipolar disorder)  5) Patients with disorders accompanied by cognitive impairment (e.g., dementia and Parkinson’s disease) or history of head trauma with loss of consciousness/convulsions  6) Individuals taking any drug that may affect sleep within 4 weeks from Visit 1  7) Individuals taking oral steroids or corticosteroids within 4 weeks from Visit 1  8) Individuals consuming health supplements to improve sleep within 4 weeks from Visit 1  9) Individuals who are sensitive or allergic to the ingredients in the product tested in the study  10) Individuals with a body mass index of ≥30 kg/m^2^ at Visit 1  11) Patients receiving inpatient care, drug therapy, or rehabilitation treatment for alcohol abuse or alcohol-induced disorder  12) Individuals with irregular sleep habits due to shift work or night work within 4 weeks from Visit 1  13) Individuals who participated or plan to participate in another interventional clinical trial within 4 weeks from Visit 1  14) Women who are pregnant or breastfeeding or planning to get pregnant during the study period  15) Individuals determined to be ineligible by the investigator for other reasons |
